# Adaptive reference update (ARU) algorithm. A stochastic search algorithm for efficient optimization of multi-drug cocktails

**DOI:** 10.1186/1471-2164-13-S6-S12

**Published:** 2012-10-26

**Authors:** Mansuck Kim, Byung-Jun Yoon

**Affiliations:** 1 Department of Electrical and Computer Engineering, Texas A&M University, College Station, TX 77843-3128, USA

## Abstract

**Background:**

Multi-target therapeutics has been shown to be effective for treating complex diseases, and currently, it is a common practice to combine multiple drugs to treat such diseases to optimize the therapeutic outcomes. However, considering the huge number of possible ways to mix multiple drugs at different concentrations, it is practically difficult to identify the optimal drug combination through exhaustive testing.

**Results:**

In this paper, we propose a novel stochastic search algorithm, called the adaptive reference update (ARU) algorithm, that can provide an efficient and systematic way for optimizing multi-drug cocktails. The ARU algorithm iteratively updates the drug combination to improve its response, where the update is made by comparing the response of the current combination with that of a reference combination, based on which the beneficial update direction is predicted. The reference combination is continuously updated based on the drug response values observed in the past, thereby adapting to the underlying drug response function. To demonstrate the effectiveness of the proposed algorithm, we evaluated its performance based on various multi-dimensional drug functions and compared it with existing algorithms.

**Conclusions:**

Simulation results show that the ARU algorithm significantly outperforms existing stochastic search algorithms, including the Gur Game algorithm. In fact, the ARU algorithm can more effectively identify potent drug combinations and it typically spends fewer iterations for finding effective combinations. Furthermore, the ARU algorithm is robust to random fluctuations and noise in the measured drug response, which makes the algorithm well-suited for practical drug optimization applications.

## Background

Biological networks are known to be redundant at various levels, which makes them robust to various types of perturbations. As a consequence, it is generally difficult to change their long-term dynamics by blocking a specific gene or intervening in a specific pathway. This is one of the reasons why monotherapy is often not very effective in treating complex diseases, such as cancer and diabetes. In fact, multi-target therapeutics based on combinatory drugs are known to be much more effective, and they are commonly used these days for treating various diseases [1-6]. However, considering the huge number of possible ways to mix multiple drugs, it is practically impossible to find the optimal "drug cocktail" simply by trial and error or by exhaustive testing. Clearly, we need a systematic way of identifying the most effective drug cocktail, and recently, several algorithms have been proposed to address the problem of combinatorial drug optimization [7-12].

For example, Calzolari et al. [7] developed a drug optimization algorithm based on sequential decoding algorithms that have been traditionally used in digital communications [13,14]. In [7], it was shown that we can algorithmically identify the optimal drug combination by testing only a relatively small number of drug combinations, compared to exhaustive search. Unlike the approach proposed by Calzolari et al. [7], which was deterministic, Wong et al. [9] proposed a different approach based on a stochastic search algorithm, called the Gur Game algorithm [15,16]. In this work [9], they formed a closed-loop optimization framework, in which the Gur Game algorithm was used to predict an updated drug combination that is likely to improve the current drug response, and the drug combination is iteratively updated until the response is maximized. It was shown that this closed-loop optimization method can quickly identify potent drug combinations. More recently, another stochastic search algorithm was proposed in [11] that addresses the limitations of the Gur Game algorithm, thereby further improving the performance of the closed-loop optimization approach originally proposed in [9].

In this paper, we propose a novel stochastic search algorithm, called the adaptive reference update (ARU) algorithm, that can significantly improve the performance of the existing stochastic search algorithms [9,11]. The key idea of this algorithm is to adaptively update the reference drug combination to guide the search algorithm and help it to make better informed guesses without requiring extensive prior knowledge of the underlying biological network. We demonstrate that the proposed ARU algorithm outperforms existing stochastic drug optimization algorithms, in terms of both efficiency, success rate, and robustness.

## Methods

### Combinatorial drug optimization problem

Suppose we have *N *different types of drugs, where each drug can take one of pre-specified concentrations. Our goal is to predict the optimal drug cocktail, by mixing the available drugs, that maximizes the overall therapeutic effect. Let *x_n _*be the concentration of the *n*-th drug, where *x_n _*can take one of *M_n _*distinct concentrations in the set $C_{n}=\left\{c_{n}^{1}\text{, }c_{n}^{2}\text{, }c_{n}^{3},\;\;.\;\;.\;\;.\;\;,\;c_{n}^{M_{n}}\right\}$, where $c_{n}^{k}<c_{n}^{k+1}$ for all *k*. The drug cocktail is represented by an *N*-dimensional vector **x **= (*x*_1_, *x*_2_, . . . , *x_N_*), which consists of the *N *drug concentrations. Let *f*(**x**) be the normalized drug response that quantitatively measures the effectiveness of a given drug combination **x**. We assume the response has been normalized such that 0≤f(x)≤1forx∈X, where $X=C_{1}\;\times \;C_{2}\;\times \;\;\cdot \cdot \cdot \;\;\times \;C_{N}$ is the set of all possible drug combinations. We denote the number of all possible drug combinations as $M=\;|X|\;=\prod_{n=1}^{N}M_{n}.\;f\left(x\right)=0$ implies that the drug cocktail **x **is completely ineffective, and larger *f *(**x**) implies higher efficacy. In practical applications, *f *(**x**) may be obtained by monitoring the cell response to a drug intervention using fluorescent imaging, microarrays, or sequencing techniques. Under this setting, we aim to find the optimal drug combination x* that maximizes the normalized drug response:

$$\underset{x\in X}{x^{*}=\text{arg}\text{max}\text{f}(x)}.$$

As we can see, this is a combinatorial optimization problem, in which we have to find the optimal drug combination out of *M*_1 _*M*_2 _. . . *M_N _*possible combinations. The total number of distinct drug combinations quickly grows as the number of drugs increases. Considering the practical cost of experimentally measuring the normalized drug response function *f *(**x**), it is apparent that we cannot test all drug combinations to find the most effective one.

### Stochastic search algorithms

Stochastic search algorithms [9,11] aim to efficiently identify the potent drug combinations without exploring the entire combinatorial solution space. The basic idea is to randomly search through the solution space by iteratively updating the drug combination until an effective combination emerges. At each step, the current drug combination is incrementally updated towards the direction that is likely to improve the overall drug response. The updating decision is made in a stochastic manner, which allows the search to proceed towards directions that are deemed to be less likely to improve the response. This is an important characteristic of stochastic search algorithms, which is critical for keeping the search from being trapped in local maxima. Since a stochastic search algorithm tries to arrive at the optimal solution (i.e., the most effective drug combination) by performing iterative local searches, its overall performance depends on how it chooses the next solution state (i.e., an updated drug combination in  X, the set of all possible combinations) from a given state (i.e., the current drug combination). The two performance metrics of interest are: (i) the effectiveness of the predicted drug combination, in terms of how close its response is to the optimal response, and (ii) the number of search steps that the algorithm needs to take until an effective combination is found. Basically, we want to predict a potent drug cocktail by testing minimal number of drug combinations to minimize the actual experimental cost for measuring the cell response to combinatorial drugs. When choosing the next state, the search algorithm has to be as parsimonious as possible, in terms of the number of function evaluations, so that the overall experimental cost for identifying the optimal drug combination can be minimized. This has been one of the main design considerations of existing stochastic search algorithms that have been developed for combinatorial drug optimization [9,11].

For example, the Gur Game algorithm adopted in [9] determines how to update the drug combination solely based on the current drug response. Suppose $x^{c}=\left(x_{1}^{c},\;x_{2}^{c},\cdots ,x_{N}^{c}\right)$ is the current drug combination with a normalized drug response of *f *(**x***^c^*). The algorithm generates *N *random numbers *r_n _*∈ [0,1], one for each drug. Each *r_n _*is compared against the current drug response $x^{c}=\left(x_{1}^{c},\;x_{2}^{c},\cdots ,x_{N}^{c}\right)$, which is used to either "reward" or "penalize" the *n*-th drug. For example, the drug is rewarded if *f *(**x***^c^*) >*r_n_*. Otherwise, it is penalized. This update process is repeated for each of the *N *drugs. According to this scheme, drug combinations with higher response are more likely to be rewarded, while combinations with lower response are more likely to be penalized. In the long run, the algorithm is expected to drive the concentration of each drug towards the one that maximizes the response. Now, an important relevant question is what is the right way of rewarding (or penalizing) the current concentration of a given drug. The Gur Game algorithm used in [9] uses a predetermined finite state automaton (FSA) for this purpose. For example, let $x^{c}=c_{n}^{k}\in C_{n}$ be the current concentration of the *n*-th drug. Suppose we have *f *(**x***^c^*) >*r_n_*, hence the current concentration $x_{n}^{c}$ of the *n*-th drug should be rewarded. The FSA in [9] is designed such that the drug concentration is increased further if the current concentration $x_{n}^{c}$ is larger than a reference concentration $c_{n}^{\text{ref}}$, and decreased further if $x_{n}^{c}$ is smaller than $c_{n}^{\text{ref}}$. More specifically, if $x_{n}^{c}=c_{n}^{k}$and *f*(**x***^c^*) >*r_n_*, then the drug concentration is updated as follows.

(1) $$x_{n}^{c}=\left\{\begin{array}{c} c_{n}^{k+1},\;\text{if }\;x_{n}^{c}>c_{n}^{\text{ref}}\text{and }k<M_{n}\\ c_{n}^{k},\;\;\text{if}\;\;x_{n}^{c}>c_{n}^{\text{ref}}\text{and }k=M_{n}\\ c_{n}^{k-1},\;\text{if }\;x_{n}^{c}<c_{n}^{\text{ref}}\text{and }k>1\\ c_{n}^{k},\;\;\text{if }\;x_{n}^{c}<c_{n}^{\text{ref}}\text{and }k=1 \end{array}\right)$$

The reference concentration $c_{n}^{\text{ref}}$ is typically chosen as the median of the set $C_{n}$. As shown in (1), the drug concentration remains unchanged if it cannot be increased (or decreased) further. Now, suppose that *f *(**x***^c^*) <*r_n_*, and therefore the current concentration $x_{n}^{c}=c_{n}^{k}$ should be penalized. In this case, the concentration is updated in the opposite direction:

(2) $$x_{n}^{c}=\left\{\begin{array}{c} c_{n}^{k-1},\;\text{if }\;x_{n}^{c}>c_{n}^{\text{ref}}\\ c_{n}^{k+1},\;\text{if }\;x_{n}^{c}<c_{n}^{\text{ref}} \end{array}\right)$$

**Figure 1:**
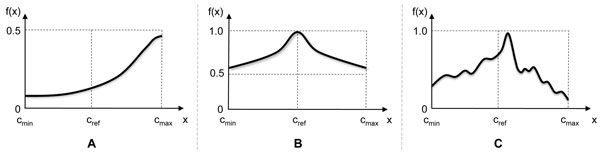
**Drug response functions**. (A) A normalized drug response *f*(*x*) that is always below 0.5. (B) A normalized drug response *f*(*x*) that is always above 0.5. (C) A drug response function with a large number of small variations.

Note that penalization moves the current drug concentration closer to the reference concentration $c_{n}^{\text{ref}}$. As previously discussed in [11], one of the weaknesses of the Gur Game algorithm is that it uses a predetermined FSA for updating (i.e., rewarding/penalizing) the drug concentrations and does not adapt to the drug response function at hand, which is not known in advance. As a result, the algorithm may perform poorly unless the drug response function *f*(**x**) is properly normalized and the reference concentration $c_{n}^{\text{ref}}$ is chosen adequately for each drug. For example, consider the one-dimensional drug response *f*(*x*) shown in Figure 1A. As we can see, the drug response has been over-normalized, hence *f*(*x*) < 0.5 for any allowed concentration *x *∈ [*c*_min_, *c*_max_]. Since *f*(*x*) < 0.5, a uniformly distributed random number *r *∈ [0,1] is more likely to be larger than *f*(*x*). This implies that the Gur Game algorithm always tends to penalize the current drug concentration (no matter what its value is), which will probabilistically drive the concentration towards *c*_ref _although it is clearly not optimal. Figure 1B shows another drug response, for which the Gur Game algorithm will not work properly. In this example, we have *f*(*x*) > 0.5, hence the Gur Game algorithm is always more likely to reward the current drug concentration, which tends to drive the concentration away from the reference concentration *c*_ref_. This will push the concentration either towards *c*_min _or *c*_max_, both of which are suboptimal.

The enhanced stochastic algorithm proposed in [11] addresses this problem by making the search algorithm adapt to a given drug response. The basic idea of this algorithm is to make an "informed-guess" about how to beneficially update a given drug concentration, instead of following a predetermined update rule. Unlike the Gur Game algorithm in [9], where all *N *drugs are simultaneously updated based on the (same) current drug response *f*(**x***^c^*), the enhanced algorithm updates the concentration of one drug at a time. As an example, suppose during the last update of the *n*-th drug, the drug combination has been updated as

$$x=\left(x_{1},\cdots \;,\cdots \;,x_{N}\right)\Rightarrow x^{\prime }\;\text{ = }\;\text{(}x_{1}^{\prime },\cdots \;,\cdots \;,x_{N}^{\prime }\text{),}$$

where **x **and $x^{\prime }$ are identical except for the concentration of the *n*-th drug, hence $x_{i}=x_{i}^{\prime }\left(i\neq n\right)$and $x_{i}\neq x_{i}^{\prime }\left(i=n\right)$. We assume that *x_n _*and $x_{n}^{\prime }$ differ only by a single concentration level, so that $x_{n}=c_{n}^{k}$and$x_{n}^{\prime }=c_{n}^{k+1}$, or $x_{n}=c_{n}^{k+1}$and $x_{n}^{\prime }=c_{n}^{k}$, for some *k*. The algorithm compares the two drug responses *f *(**x**) and $f\left(x^{\prime }\right)$, thereby determine wether it would be more beneficial to further increase or decrease the concentration of the *n*-th drug. For example, we may have the following four cases.

(3) $$\begin{array}{c} \left(\text{Case - 1}\right)\;x_{n}<{x^{\prime }}_{n}\;\text{and}\;f\left(x\right)<f\left(x^{\prime }\right):increasing\text{ the concentration is more beneficial}\\ \left(\text{Case - }2\right)\;x_{n}>{x^{\prime }}_{n}\;\text{and }f\left(x\right)>f\left(x^{\prime }\right):increasing\text{ the concentration is more beneficial}\\ \left(\text{Case - }3\right)\;x_{n}<{x^{\prime }}_{n}\;\text{and }f\left(x\right)>f\left(x^{\prime }\right):decreasing\text{ the concentration is more beneficial}\\ \left(\text{Case - }4\right)\;x_{n}>{x^{\prime }}_{n}\text{ and }f\left(x\right)<f\left(x^{\prime }\right):decreasing\text{ the concentration is more beneficial}. \end{array}$$

The above rules allow the algorithm to adaptively determine *how *to reward (or penalize) a given drug concentration based on the observed drug response values. However, the decision *whether *to reward or penalize the current drug is made in a probabilistic manner. For this purpose, we evaluate the following function

(4) $$g\left(x,\;x^{\prime }\right)=\frac{1}{2}\left\{1+\alpha \cdot \text{max}\left[f\left(x\right),f\left(x^{\prime }\right)\right]\right\},$$

where *α *∈ [0, 1] is a control parameter that adjusts the randomness of the algorithm [11]. This *g*(**x**, **x'**) is compared with a uniformly distributed random number *r_n _*∈ [0, 1]. If *g*(**x**, **x'**) *> r_n_*, the *n*-th drug is rewarded, i.e., updated in such a way that appears to be more beneficial for enhancing the drug response according to the rules shown in (3). Otherwise, the *n*-th drug is penalized, i.e., updated in a way that appears to be less beneficial based on the past observations. It is not difficult to see that this algorithm is always more likely to reward, or beneficially update, a given drug. Since the algorithm proposed in [11] adaptively determines how to update the drug concentration based on previous observations, it can also effectively deal with drug response functions shown in Figures 1A and 1B, for which the Gur Game algorithm does not perform well. Despite its merits, this algorithm also has its own shortcomings. For example, as the update rule for a given drug is determined only based on the two observations that correspond to its latest update, not on a longer-range trend, the algorithm may be sensitive to small variations in the drug response. As a result, it may not show satisfactory search performance for drug response functions that are similar to the one in Figure 1C. Furthermore, considering that *f*(**x**) has to be experimentally estimated, where a certain level of measurement noise and small variations due to a number of practical factors may not be avoidable, such sensitivity may adversely affect the overall performance of the algorithm. Another weakness of the algorithm is that it only utilizes a very small part of the past observations without fully utilizing them. In the following section, we introduce a novel stochastic search algorithm that can effectively address the aforementioned issues.

### The adaptive reference update (ARU) stochastic search algorithm

**Figure 2:**
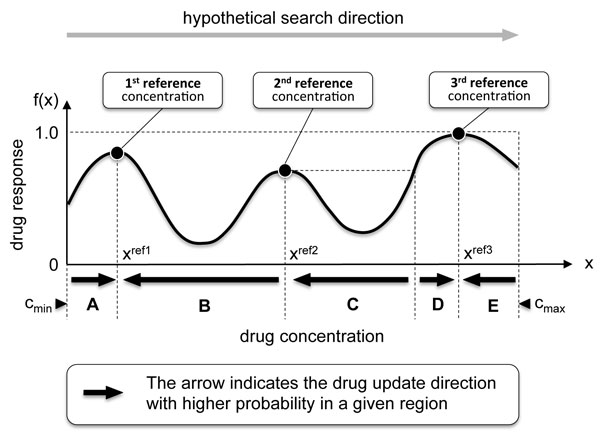
**Updating the reference point**. As the search for the optimal drug concentration continues from left to right (from the lowest concentration to the highest one), the reference concentration is updated from the initial drug concentration *c*_min _to the local maximum points *x*^ref1^, *x*^ref2^, and *x*^ref3^, according to this order.

In order to make the search algorithm robust to small variations in the observed drug response, the update rules have to be decided based on the general trend of the drug response change over a wide range of drug concentration, not just based on the response change resulting from a *single-level *concentration change. Based on this motivation, we propose a novel algorithm called the **adaptive reference update (ARU) algorithm**. In this algorithm, we compare the current drug response *f*(**x***^c^*) with the response *f*(**x**^ref^) of a *reference drug combination ***x**^ref^, which is adaptively updated based on past observations. In the beginning, **x**^ref ^is set to the initial drug concentration, where we start the search process. During the search, when the algorithm encounters a local maximum, the current reference combination is replaced by the corresponding drug combination. As an example, let us consider the one-dimensional drug response function *f*(*x*) in Figure 2. For illustration, we consider the following hypothetical search process, where the drug concentration is constantly updated from left to right, starting from the lowest concentration *c*_min _to the highest concentration *c*_max_. Suppose the search begins at the concentration *x *= *c*_min_. Initially the reference concentration is also set to this initial drug concentration *x*^ref ^← *c*_min_. As the search reaches the first local maximum, the reference concentration is updated to this local maximum solution *x*^ref ^← *x*^ref1^. As the search continues to the right, this reference concentration is used until we reach the next local maximum. After passing the second local maximum solution *x*^ref2^, the reference point is updated to *x*^ref ^← *x*^ref2^. In a similar manner, as the search continues further to the right, the reference point is updated to *x*^ref ^← *x*^ref3 ^after passing the third local maximum point.

At each iteration, the current drug response *f*(**x***^c^*) is compared to the response *f*(**x**^ref^) of the reference drug combination, based on which the drug update rule is determined. For example, let $x^{c}=\left(\cdots ,x_{n}^{c},\;\cdots \;\right)$ and $x^{\text{ref}}=\left(\cdots ,x_{n}^{\text{ref}},\;\cdots \;\right)$, and assume that we want to update the concentration of the *n*-th drug by comparing the two drug response values *f*(**x***^c^*) and *f*(**x**^ref^). As before, we have the following four possible cases.

(5) $$\begin{array}{c} \left(\text{Case}-1\right)\;x_{n}^{c}<x_{n}^{\text{ref}}\;\text{and }f\left(x^{c}\right)<f\left(x^{\text{ref}}\right):increasing\text{ the concentration is more beneficial}\\ \left(\text{Case}-2\right)\;x_{n}^{c}>x_{n}^{\text{ref}}\;\text{and }f\left(x^{c}\right)>f\left(x^{\text{ref}}\right):increasing\text{ the concentration is more beneficial}\\ \left(\text{Case}-3\right)\;x_{n}^{c}<x_{n}^{\text{ref}}\;\text{and }\;f\left(x^{c}\right)>f\left(x^{\text{ref}}\right):decreasing\text{ the concentration is more beneficial}\\ \left(\text{Case}-4\right)\;x_{n}^{c}>x_{n}^{\text{ref}}\;\text{and}\;f\left(x^{c}\right)<f\left(x^{\text{ref}}\right):decreasing\text{ the concentration is more beneficial}. \end{array}$$

Conceptually, we can view the above as estimating the "virtual" slope between two points $\left(x_{n}^{c},\;f\left(x^{c}\right)\right)$ and $\left(x_{n}^{\text{ref}},\;f\left(x^{\text{ref}}\right)\right)$ as follows

(6) $$\frac{f\left(x^{\text{ref}}\right)-f\left(x^{c}\right)}{x_{n}^{\text{ref}}-x_{n}^{c}},$$

based on which we determine how to update the concentration *x_n _*of the *n*-th drug to increase the drug response *f*( ). Given the update rules in (5), the actual update decision is made by evaluating the following function

(7) $$g\left(x^{c},\;x^{\text{ref}}\right)=\frac{1}{2}\left\{1+\alpha \cdot \text{max}\left[f\left(x^{c}\right),f\left(x^{\text{ref}}\right)\right]\right\}$$

and comparing it with a random number *r_n _*∈ [0, 1]. If *g*(**x***^c^*, **x**^ref^) >*r_n_*, the drug concentration *x_n _*is updated by a single level, following the beneficial update direction predicted by (5). Otherwise, the concentration is updated in the opposite direction. As briefly mentioned before, the parameter *α *∈ [0, 1] controls the randomness of the algorithm. For example, *α *= 0 will make the search process completely random, regardless of the observed drug responses. Using a larger *α *means that we are giving a larger weight to the past observations when deciding how to update the drug concentrations. The value of this control parameter is limited to *α *≤ 1 such that *g*(**x***^c^*, **x**^ref^) ≤ 1. Also note that we always have *g*(**x***^c^*, **x**^ref^) ≥ 0.5, which implies that at any drug update step, the update is always more likely to take place in accordance with the rules in (5), which have been derived based on past observations of the drug response. In other words, the ARU algorithm tries to effectively utilize the past response data to beneficially update the drug concentrations, and ultimately, to identify a potent drug combination, while keeping the search still stochastic. For illustration, let us again consider the drug response function in Figure 2, where the hypothetical search process proceeds from the lowest drug concentration to the highest concentration. The black solid arrows below the graph shows the drug update direction that gets higher probability according to the new algorithm, described above. For example, in region-A (*c*_min _*< × < x*^ref1^), the algorithm tends to increase the drug concentration *x *further, as the response *f*(*x*) is larger than *f*(*c*_min_) of the initial reference concentration (i.e., *c*_min_). As *x *continues to increase and passes the first local maximum point *x*^ref1^, the reference is updated to *x*^ref ^← *x*^ref1^. In region-B (*x*^ref1 ^*< × < x*^ref2^), the search algorithm tends to drive the concentration towards *x*^ref1 ^by decreasing the concentration. Suppose the search continues to increase the drug concentration *x *beyond *x*^ref2^, the second local maximum point, despite the tendency of the algorithm to decrease *x *back to *x*^ref1^. After passing *x*^ref2^, the reference is updated to *x*^ref ^← *x*^ref2^. In region-C, the search algorithms assigns higher probability to the update rule that tries to bring the concentration down to *x*^ref2^, since *f*(*x*) *< f*(*x*^ref2^) in the given region. However, once *x *enters region-D, where *f*(*x*) *> f*(*x*^ref2^), the algorithm begins to drive the drug concentration *x *further to the right until it passes the third local maximum point *x*^ref3^. The reference concentration is updated to *x*^ref ^← *x*^ref3^, once the search continues to the right and the drug concentration *x *gets larger than *x*^ref3^. Since *f*(*x*^ref3^) is larger than *f*(*x*) in region-D (*x*^ref3 ^*< × < c*_max_), the search algorithm will tend to bring the concentration down to the current reference concentration, namely, *x*^ref ^= *x*^ref3^.

Choosing a local maximum solution as a reference combination has a number of practical advantages. First of all, it allows the algorithm to adjust the drug update rules based on a long-range trend of the given drug response function, which makes the algorithm robust to small variations in the observed response. Another advantage of using a long-range trend is that the search process will become also less sensitive to random fluctuations that may exist in the observed drug response. Considering that the drug response function *f*(**x**) has to be experimentally estimated through actual biological experiments, where random factors (e.g., measurement noise) that affect the estimation results cannot be completely ruled out, such robustness is critical for the algorithm to be used in practical drug optimization applications. It is also beneficial to use the drug combination that corresponds to the most recent local maximum response, instead of the drug combination that has yielded the highest response among all past combinations, as the reference point. This prevents the search process from dwelling too much on past observations, while keeping it robust to variations and random fluctuations.

### Drug response functions

In order to evaluate the overall performance of the ARU algorithm, we used the algorithm to search for the optimal drug cocktail for several different drug response functions.

**Figure 3:**
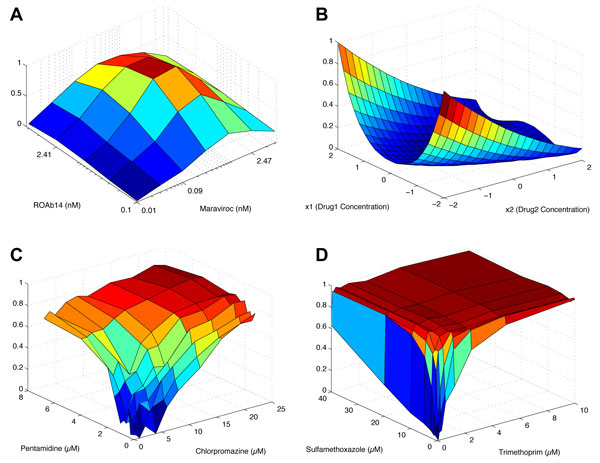
**Two-dimensional drug response functions**. (A) Inhibition of HIV. (B) Second De Jong function (Rosenbrock's saddle). (C) Inhibition of A549 lung carcinoma cell proliferation. (D) Inhibition of bacteria (*S*. *aureus*) proliferation.

**Two-dimensional drug response functions **For performance assessment, we first used the four two-dimensional drug response functions that are shown in Figure 3. The first drug response function *f*_2*a*_(*x*_1_, *x*_2_) shown in Figure 3A is the normalized HIV inhibitor response obtained from [17], where *x*_1 _∈ {0, 0.01, 0.03, 0.09, 0.27, 0.82, 2.47, 7.41, 22.22, 66.67}(*nM*) was considered for Maraviroc and *x*_2 _∈ {0, 0.09, 0.27, 0.8, 2.41, 7.22, 21.67, 65}(*nM*) for ROAb14. The second drug response *f*_2*b*_(*x*_1_, *x*_2_) shown in Figure 3B is the normalized second De Jong function (Rosenbrock's saddle) [18]. We considered *x*_1_, *x*_2 _∈ {*c*_0_, *c*_1_, ... , *c*_20_}, where *c_k _*= 4(*k/*20 - 0.5), obtained by evenly dividing the range [-2, 2] into 21 distinct values. The third drug response function *f*_2*c*_(*x*_1_, *x*_2_) in Figure 3C is the normalized lung cancer inhibition response obtained from [1], where *x*_1 _∈ {0, 1, 2, 4, 6, 8, 12, 16, 20, 22}(*μM *) was considered for Chlorpromazine and *x*_2 _∈ {0, 0.25, 0.4, 0.6, 0.8, 1, 1.5, 2, 4, 6.8}(*μM *) for Pentamidine. Finally, Figure 3D shows the fourth response function *f*_2*d*_(*x*_1_, *x*_2_), the normalized bacterial (*S. aureus*) inhibition response reported in [6]. *x*_1 _∈ {0, 0.08, 0.16, 0.32, 0.63, 1.25, 2.5, 5, 10} was considered for Trimethoprim and *x*_2 _∈ {0, 0.31, 0.62, 1.25, 2.5, 5, 10, 20, 40} was considered for Sulfamethoxazole. All four drug response functions were normalized to span the entire range [0, 1], such that the minimum response is 0 and the maximum response is 1.

**Multi-dimensional drug response functions **To evaluate the performance for optimizing multi-drug cocktails, we defined several hypothetical drug response functions with up to six drugs. First, we defined two 3-dimensional drug functions *f*_3*a*_(*x*_1_, *x*_2_, *x*_3_) and *f*_3*b*_(*x*_1_, *x*_2_, *x*_3_). The first function is defined as

(8) $$f_{3a}\left(x_{1},\;x_{2},\;x_{3}\right)=x_{1}^{2}\cdot {\text{sin}}^{2}\left(x_{2}\right)\cdot {\text{cos}}^{2}\left(x_{3}\right),$$

where each of *x*_1_, *x*_2_, *x*_3 _can take one of the 11 discrete concentrations that evenly divide the range [-2.5, 2.5]. The second function is defined as follows

(9) $$f_{3b}\left(x_{1},\;x_{2},\;x_{3}\right)=peaks\left(x_{1},\;x_{2}\right)\cdot x_{3},$$

using the Matlab peaks(*x*_1_, *x*_2_) function. We assume that each drug can take one of the 11 discrete values that evenly divide [-3, 3]. Next, we defined two 4-dimensional drug functions

(10) $$f_{4a}\left(x_{1},\;x_{2},\;x_{3},\;x_{4}\right)=x_{1}\cdot e^{-\left(x_{1}^{2}+x_{2}^{2}+x_{3}^{2}+x_{4}^{2}\right)}$$

and

(11) $$f_{4b}\left(x_{1},\;x_{2},\;x_{3},\;x_{4}\right)={\text{cos}}^{2}\left(0.3x_{1}\right)\cdot \text{sin}\left(0.3x_{2}\right)\cdot \text{tan}\left(0.1x_{3}\right)\cdot x_{4}.$$

We assume that *x*_1 _and *x*_2 _in the first function *f*_4*a*_(*x*_1_, *x*_2_, *x*_3_, *x*_4_) can take one of the 11 discrete values that evenly divide the range [-2, 2], while *x*_3 _and *x*_4 _can take one of the 11 discrete values that evenly divide the range [-3, 3]. For the second drug response function *f*_4*b*_(*x*_1_, *x*_2_, *x*_3_, *x*_4_), we assume that each drug can take one of the 11 distinct values that evenly divide [-3, 3]. In addition, we also defined the following 5-dimensional drug response functions

(12) $$f_{5a}\left(x_{1},\;x_{2},\;x_{3},\;x_{4},\;x_{5}\right)=e^{-x_{1}}\cdot {\text{cos}}^{2}\left(x_{2}\right)\cdot x_{3}^{2}\cdot \left[e^{-{\left(x_{4}+2\right)}^{2}-{\left(x_{5}+3\right)}^{2}}+e^{-{\left(x_{4}-2\right)}^{2}-{\left(x_{5}-3\right)}^{2}}\right]$$

and

(13) $$f_{5b}\left(x_{1},\;x_{2},\;x_{3},\;x_{4},\;x_{5}\right)=\frac{1}{2}\text{peaks}\left(x_{1},\;x_{2}\right)\cdot \text{cos}\left(0.5x_{3}\right)\cdot \text{sin}\left(0.5x_{4}\right)\cdot x_{5}^{2}.$$

For the first function *f*_5*a*_(*x*_1_, *x*_2_, *x*_3_, *x*_4_, *x*_5_), *x*_1 _and *x*_2 _are allowed to take any value from the set of values obtained by evenly dividing the range [-2, 2] into 11 discrete concentrations. The remaining drug concentrations (*x*_3_, *x*_4_, and *x*_5_) can take any of the 11 concentrations that evenly divide [-4.5, 4.5]. For the second drug response function *f*_5*b*_(*x*_1_, *x*_2_, *x*_3_, *x*_4_, *x*_5_), we assume that each drug can take one of the 11 discrete values that evenly divide [-3, 3]. Finally, we also defined two 6-dimensional drug response functions

(14) $$f_{6a}\left(x_{1},\;x_{2},\;x_{3},\;x_{4},\;x_{5},\;x_{6}\right)=e^{-0.75\left(x_{1}\right)}\cdot \left({\text{sin}}^{2}\left(x_{2}\right)+\text{cos}\left(x_{3}\right)\right)\cdot e^{-0.75\left(x_{4}^{2}+x_{5}^{2}\right)}\cdot x_{6}$$

and

(15) $$f_{6b}\left(x_{1},\;x_{2},\;x_{3},\;x_{4},\;x_{5},\;x_{6}\right)=e^{-0.1\left(x_{1}^{2}-x_{2}^{2}\right)-0.1\left(x_{3}^{2}+x_{4}^{2}\right)}\cdot {\text{cos}}^{2}\left(0.2x_{5}^{3}\right)\cdot \text{sin}\left(0.2x_{6}^{3}\right),$$

where every drug concentration can take its value from one of the 11 discrete concentrations that evenly divide the range [-2.5, 2.5].

## Results

### Optimizing the combination of two drugs

We first evaluated the overall performance of the ARU stochastic search algorithm based on four two-dimensional drug response functions (see Methods). (i) HIV inhibitor response *f*_2*a*_(*x*_1_, *x*_2_), (ii) second De Jong function (Rosenbrock's saddle) *f*_2*b*_(*x*_1_, *x*_2_), (iii) normalized lung cancer inhibition response *f*_2*c*_(*x*_1_, *x*_2_), and (iv) bacterial (*S. aureus*) inhibition response *f*_2*d*_(*x*_1_, *x*_2_). These functions are shown in Figure 3. For each drug response function, we searched for the optimal drug response using the proposed algorithm, starting from randomly selected drug concentrations *x*_1 _and *x*_2_. The parameter *α *that controls the randomness of the search was set to *α *= 1, which implies that the algorithm adaptively determines the search direction (i.e., how to update the current drug concentration) by fully utilizing the past observations. Note that setting the parameter to *α *= 0 in (7) would make the search completely random and independent of the past observations: at each step, the concentration of a given drug will be randomly increased or decreased with equal probability, regardless of the update rules given in (5). In each search experiment, the iterations were repeated up to two times the total number of possible drug combinations. This experiment was repeated 5,000 times to obtain reliable results. For comparison, we performed similar experiments using the Gur Game algorithm [9] and the stochastic search algorithm proposed in [11] with *α *= 1. We computed the following two performance metrics: the success rate and the average number of unique drug combinations that need to be tested. The first metric is defined as the relative proportion of experiments, in which the algorithm was able to identify a potent drug combination whose response is within 5% of the maximum response, i.e., *f*(*x*_1_, *x*_2_) ≥ 0.95. The second metric is defined as the average number of unique drug combinations that have to be tested until a potent drug combination is identified, in case the search is successful. These performance assessment results are summarized in Table 1. Note that the Gur Game algorithm has been tested using both the simultaneous update strategy as well as the sequential update strategy. Unlike the ARU algorithm and the search algorithm proposed in [11], which update one drug at a time (i.e., "sequential" drug update), the original Gur Game algorithm adopted in [9] updates all drugs simultaneously. However, it is also possible to use the sequential update strategy with the Gur Game algorithm, and we have evaluated both strategies for comparison. Table 1 shows that the proposed ARU algorithm outperforms the existing algorithms in terms of success rate. Furthermore, when the success rates are comparable, the ARU algorithm can in general identify an effective drug combination more efficiently, as reflected in the smaller number of unique drug combinations that need to be tested. We can get a more complete picture of the efficiency of the ARU algorithm from Figure S1 and Figure S2, which respectively show the distribution of the number of unique drug combinations that need to be tested and the distribution of the number of iterations that are needed to identify a potent drug combination (see Additional file 1).

**Table 1:** Performance for optimizing the combination of two drugs.

		Gur Game (simultaneous)		Gur Game (sequential)		**Previous search algorithm **[11]**(*α *= 1)**		ARU algorithm (proposed) (*α *= 1)	
		
		success rate	**unique comb**.	success rate	**unique comb**.	success rate	**unique comb**.	success rate	**unique comb**.
*f*_2*a*_(**x**) : HIV INHIBITION	(*M *= 80)	97%	13.2	95%	17.2	100%	13.4	**100%**	**12.1**
*f*_2*b*_(**x**) : DEJONG (2ND)	(*M *= 441)	9%	3.6	10%	4.5	99%	56.2	**99%**	**46.2**
*f*_2*c*_(**x**) : CANCER INHIBITION	(*M *= 100)	58%	11.3	53%	13.0	98%	13.2	**98%**	**12.4**
*f*_2*d*_(**x**): BACTERIA INHIBITION	(*M *= 81)	96%	5.9	91%	6.8	100%	4.8	**100%**	**4.5**

### Optimizing multi-drug cocktails

Next, we tested the performance of the ARU algorithm for optimizing multi-drug cocktails that consist of three to six drugs. For this purpose, we used the eight hypothetical drug response functions that were defined before (see Methods). As in our previous experiments, for each drug response function, we used the proposed ARU algorithm (with *α *= 1) to search for a potent drug combination whose response is within 5% of the maximum response (i.e., *f*(**x**) ≥ 0.95). In each search experiment, we started from an randomly selected initial concentrations, and continued the search up to 1,000 steps for 3 drugs, 2,000 steps for 4 drugs, 3,000 steps for 5 drugs, and 4,000 steps for 6 drugs. This experiment was repeated 5,000 times to obtain reliable performance assessment results. The simulation results are summarized in Table 2. As shown in this table, the proposed algorithm boasts a significantly higher success rate compared to the Gur Game algorithm [9]. It also results in either comparable or slightly improved success rate compared to the previous drug optimization algorithm (*α *= 1) [11]. However, we can see that the ARU algorithm clearly outperforms the previous search algorithm in terms of efficiency, as reflected in the significantly smaller number of unique drug combinations that need to be tested until an effective combination is identified. Figures S3 and S5 show the distribution of the number of unique drug combinations that need to be tested to identify an effective drug cocktail (see Additional file 1). Similarly, Figures S4 and S6 show the distribution of the number of search iterations that are needed by each algorithm.

**Table 2:** Performance for optimizing multi-drug cocktails.

		Gur Game (simultaneous)		Gur Game (sequential)		**Previous search algorithm **[11]**(*α *= 1)**		ARU algorithm (proposed) (*α *= 1)	
		
		success rate	**unique comb**.	success rate	**unique comb**.	success rate	**unique comb**.	success rate	**unique comb**.
*f*_3*a*_(**x**)	(*M *= 11^3^)	1%	4.3	2%	5.5	100%	105.3	**100%**	**74.0**
*f*_3*b*_(**x**)	(*M *= 11^3^)	83%	229.4	58%	204.8	100%	88.5	**100%**	**79.4**
*f*_4*a*_(**x**)	(*M *= 11^4^)	20%	823.9	11%	666.6	100%	177.9	**100%**	**136.8**
*f*_4*b*_(**x**)	(*M *= 11^4^)	52%	706.7	24%	520.9	100%	117.9	**100%**	**91.6**
*f*_5*a*_(**x**)	(*M *= 11^5^)	8%	2.1	2%	4.8	100%	138.1	**100%**	**80.6**
*f*_5*b*_(**x**)	(*M *= 11^5^)	89%	1013.4	54%	976.2	100%	252.9	**100%**	**216.8**
*f*_6*a*_(**x**)	(*M *= 11^6^)	90%	1269.1	44%	1260.8	100%	191.9	**100%**	**178.1**
*f*_6*b*_(**x**)	(M = 11^6^)	90%	446.7	40%	1033.2	100%	238.1	**100%**	**190.1**

### Drug optimization in the presence of measurement noise

In order to use a drug optimization algorithm in practical applications, the algorithm has to be robust to random fluctuations in the estimated drug response. To evaluate the robustness of the proposed ARU algorithm, we evaluated its search performance in the presence of measurement noise and compared it with other existing stochastic search algorithms. In these experiments, we considered two different types of search strategies. In the first search strategy (referred as **type-A**), when the search algorithm happens to revisit a drug combination that was previously tested, it does *not *re-evaluate the drug response and simply uses the previously estimated value. On the other hand, according to the second strategy (referred as **type-B**), the search algorithm always re-evaluates the drug response, even if it revisits a previously evaluated drug combination, since the measured response may be different every time due to the random measurement noise. The first strategy may be useful when the noise level is relatively low, in which case this strategy may be able to reduce the total number of drug response evaluations, thereby reducing the overall experimental cost for identifying a potent drug combination. However, when the noise level is high, the search performance may be degraded as the search algorithm clings to the past (noisy) response, once it has been measured. In contrast, the second search strategy generally requires a relatively larger number of drug response evaluations, but it tends to be more robust to random fluctuations and noise in the measured drug response function.

In order to evaluate the performance of the different search algorithms in the presence of noise, we performed similar search experiments as before at three different levels of additive noise. 2%, 5%, and 8%. More precisely, we assume that

$$f_{\text{obs}}\left(x\right)=f_{\text{true}}\left(x\right)+\eta ,$$

where *f*_obs_(**x**) is the observed drug response, *f*_true_(**x**) is the true response, and *η *is an independent random noise that is uniformly distributed over (-*u*, *u*), where *u *∈ {0.02, 0.05, 0.08}. For each drug response function and a given noise level *u*, we tested the performance of both search strategies. For **type-A **search, we evaluated the success rate and the average number of unique drug combinations that have to be tested until a potent drug combination is identified. For **type-B **search, we evaluated the success rate and the average number of iterations, instead of the number of unique drug combinations, until an effective combination is identified. This is because, in a **type-B **search, the search algorithm re-evaluates the drug response even if it revisits the same drug combination that was previously tested. The simulation results are shown in Table 3,4,5,6,7, for drug response functions with two to six drugs. The parameter *α *was set to *α *= 1 for the ARU algorithm as well as the previous search algorithm proposed in [11].

**Table 3:** Performance for optimizing the combination of two drugs in the presence of noise.

	Noise level	Search type	Performance metric	Gur Game (simultaneous)	Gur Game (sequential)	**Previous search algorithm **[11]**(*α *= 1)**	ARU algorithm (proposed) (*α *= 1)
*f*_2*a*_(**x**)	**(2%)**	A	success rate unique comb.	97%	96%	100%	**100%**
				12.5	17.0	13.3	**11.6**
		B	success rate iterations	97%	95%	100%	**100%**
				37.8	45.2	25.8	**20.0**
	
	**(5%)**	A	success rate unique comb.	97%	96%	100%	**100%**
				12.6	17.1	13.3	**11.8**
		B	success rate iterations	97%	95%	100%	**100%**
				38.0	45.4	26.6	**20.2**
	
	**(8%)**	A	success rate unique comb.	97%	96%	100%	**100%**
				12.6	17.0	13.3	**12.0**
		B	success rate iterations	97%	95%	100%	**100%**
				38.2	45.4	26.8	**20.4**

*f*_2*b*_(**x**)	**(2%)**	A	success rate unique comb.	10%	10%	99%	**99%**
				3.9	4.2	57.0	**45.1**
		B	success rate iterations	10%	10%	99%	**99%**
				4.0	44	148.5	**120.0**
	
	**(5%)**	A	success rate unique comb.	9%	9%	98%	**98%**
				4.1	4.5	62.9	**52.2**
		B	success rate iterations	9%	9%	98%	**99%**
				4.3	4.7	172.1	**143.8**
	
	**(8%)**	A	success rate unique comb.	8%	9%	97%	**98%**
				4.1	4.7	66.2	**55.6**
		B	success rate iterations	9%	9%	97%	**98%**
				4.4	4.9	198.1	**167.3**

*f*_2*c*_(**x**)	**(2%)**	A	success rate unique comb.	61%	54%	98%	**98%**
				10.9	12.7	13.1	**12.5**
		B	success rate iterations	60%	54%	98%	**98%**
				33.1	36.0	35.9	**35.2**
	
	**(5%)**	A	success rate unique comb.	71%	66%	98%	**98%**
				11.4	13.2	12.9	**12.4**
		B	success rate iterations	60%	54%	98%	**98%**
				33.2	35.4	36.7	**36.0**
	
	**(8%)**	A	success rate unique comb.	88%	83%	98%	**98%**
				11.7	13.4	12.6	**12.0**
		B	success rate iterations	6.%	54%	98%	**98%**
				33.4%	34.3	37.4	**37.0**

*f*_2*d*_(**x**)	**(2%)**	A	success rate unique comb.	100%	100%	100%	**100%**
				4.9	6.0	4.6	**4.1**
		B	success rate iterations	96%	9.%	100%	**100%**
				18.3	19.7	8.2	**7.7**
	
	**(5%)**	A	success rate unique comb.	100%	100%	100%	**100%**
				4.9	5.7	4.3	**4.1**
		B	success rate iterations	96.%	91%	100%	**100%**
				18.4	19.6	8.1	**7.5**
	
	**(8%)**	A	success rate unique comb.	100%	100%	100%	**100%**
				4.8	5.6	4.4	**4.2**
		B	success rate iterations	96%	91%	100%	**100%**
				18.6	19.6	8.1	**7.1**

**Table 4:** Performance for optimizing the combination of three drugs in the presence of noise.

	Noise level	Search type	Performance metric	Gur Game (simultaneous)	Gur Game (sequential)	**Previous search algorithm **[11]**(*α *= 1)**	ARU algorithm (proposed) (*α *= 1)
*f*_3*a*_(**x**)	**(2%)**	A	success rate unique comb.	1%	3%	99%	**100%**
				2.4	7.3	110.6	**77.3**
		B	success rate iterations	1%	3%	99%	**100**%
				2.8	10.9	201.1	**139.6**
	
	**(5%)**	A	success rate unique comb.	1%	3%	99%	**100%**
				2.4	7.4	111.7	**78.4**
		B	success rate iterations	1%	3%	99%	**100**%
				2.5	10.1	201.6	**144.0**
	
	**(8%)**	A	success rate unique comb.	1%	3%	99%	**100%**
				2.5	7.7	113.3	**80.5**
		B	success rate iterations	1%	3%	99%	**100**%
				2.3	9.4	210.9	**151.7**

*f*_3*b*_(**x**)	**(2%)**	A	success rate unique comb.	86%	69%	99%	**99%**
				224.3	201.6	110.8	**93.3**
		B	success rate iterations	83%	59%	99%	**99**%
				367.1	419.1	211.5	**205.3**
	
	**(5%)**	A	success rate unique comb.	89%	72%	99%	**99%**
				222.3	201.6	116.6	**106.2**
		B	success rate iterations	83%	59%	98%	**98**%
				359.3	439.9	225.5	**222.9**
	
	**(8%)**	A	success rate unique comb.	90%	74%	97%	**98%**
				225.9	200.9	126.4	**114.3**
		B	success rate iterations	82%	60%	97%	**98**%
				359.4	431.3	249.1	**246.8**

**Table 5:** Performance for optimizing the combination of four drugs in the presence of noise.

	Noise level	Search type	Performance metric	Gur Game (simultaneous)	Gur Game (sequential)	**Previous search algorithm **[11]**(*α *= 1)**	ARU algorithm (proposed) (*α *= 1)
*f*_4*a*_(**x**)	**(2%)**	A	success rate unique comb.	21%	13%	96%	**98%**
				798.9	711.7	393.9	**327.4**
		B	success rate iterations	21%	12%	96%	**97**%
				941.9	1032.1	558.3	**452.3**
	
	**(5%)**	A	success rate unique comb.	21%	14%	90%	**95%**
				816.3	653.6	473.1	**398.3**
		B	success rate iterations	21%	13%	90%	**95%**
				895.6	1022.9	675.4	**581.2**
	
	**(8%)**	A	success rate unique comb.	24%	14%	85%	**95%**
				858.7	681.6	505.7	**433.6**
		B	success rate iterations	23%	13%	84%	**92%**
				997.1	1008.9	720.8	**648.5**

*f*_4*b*_(**x**)	**(2%)**	A	success rate unique comb.	62%	41%	100%	**100%**
				634.5	523.1	138.0	**103.1**
		B	success rate iterations	51%	26%	100%	**100%**
				932.4	903.0	236.9	**182.8**
	
	**(5%)**	A	success rate unique comb.	75%	68%	100%	**100%**
				610..2	468.0	231.1	**150.9**
		B	success rate iterations	50%	25%	100%	**100%**
				855.9	921.2	411.0	**258.8**
	
	**(8%)**	A	success rate unique comb.	86%	82%	98%	**100%**
				525.8	430.1	314.2	**215.9**
		B	success rate iterations	50%	24%	94%	**100%**
				835.3	979.2	602.0	**393.8**

**Table 6:** Performance for optimizing the combination of five drugs in the presence of noise.

	Noise level	Search type	Performance metric	Gur Game (simultaneous)	Gur Game (sequential)	**Previous search algorithm **[11]**(*α *= 1)**	ARU algorithm (proposed) (*α *= 1)
*f*_5*a*_(**x**)	**(2%)**	A	success rate unique comb.	8%	9%	100%	**100%**
				2.1	4.4	139.3	**122.5**
		B	success rate iterations	9%	11%	100%	**100%**
				2.1	6.0	172.4	**154.9**
	
	**(5%)**	A	success rate unique comb.	9%	11%	100%	**100%**
				3.9	7.9	142.1	**129.1**
		B	success rate iterations	9%	12%	100%	**100%**
				108.3	38.6	177.1	**155.6**
	
	**(8%)**	A	success rate unique comb.	10%	13%	100%	**100%**
				7.1	20.2	144.5	**131.4**
		B	success rate iterations	9%	13%	100%	**100%**
				191.4	70.8	182.3	**156.5**

*f*_5*b*_(**x**)	**(2%)**	A	success rate unique comb.	89%	55%	100%	**100%**
				917.9	1026.3	407.1	**343.9**
		B	success rate iterations	90%	55%	100%	**100%**
				999.8	1325.1	516.5	**444.3**
	
	**(5%)**	A	success rate unique comb.	90%	59%	97%	**98%**
				932.8	1002.7	507.7	**463.6**
		B	success rate iterations	90%	56%	99%	**99%**
				1004.7	1332.7	656.9	**562.4**
	
	**(8%)**	A	success rate unique comb.	91%	59%	97%	**98%**
				959.5	971.2	578.8	**534.8**
		B	success rate iterations	90%	56%	99%	**99%**
				1015.2	1341.0	735.0	**668.6**

**Table 7:** Performance for optimizing the combination of six drugs in the presence of noise.

	Noise level	Search type	Performance metric	Gur Game (simultaneous)	Gur Game (sequential)	**Previous search algorithm **[11]**(*α *= 1)**	ARU algorithm (proposed) (*α *= 1)
*f*_6*a*_(**x**)	**(2%)**	A	success rate unique comb.	91%	43%	100%	**100%**
				1280.0	1214.1	476.8	**432.6**
		B	success rate iterations	90%	43%	100%	**100%**
				1352.6	1662.6	531.4	**503.6**
	
	**(5%)**	A	success rate unique comb.	90%	44%	99%	**99%**
				1262.3	1247.2	621.0	**598.3**
		B	success rate iterations	90%	45%	100%	**100%**
				1396.8	1675.9	763.7	**736.1**
	
	**(8%)**	A	success rate unique comb.	90%	46%	98%	**98%**
				1204.7	1302.4	723.2	**698.8**
		B	success rate iterations	90%	46%	98%	**98%**
				1412.2	1681.9	875.2	**834.3**

*f*_6*b*_(**x**)	**(2%)**	A	success rate unique comb.	91%	43%	100%	**100%**
				509.9	971.0	341.4	**237.7**
		B	success rate iterations	94%	44%	100%	**100%**
				1240.7	1646.0	436.5	**293.5**
	
	**(5%)**	A	success rate unique comb.	90%	42%	100%	**100%**
				473.8	970.7	349.9	**279.8**
		B	success rate iterations	94%	44%	100%	**100%**
				1278.6	1704.9	480.8	**324.6**
	
	**(8%)**	A	success rate unique comb.	89%	42%	100%	**100%**
				454.4	969.9	457.4	**353.2**
		B	success rate iterations	94%	44%	100%	**100%**
				1333.1	1775.5	545.3	**391.5**

As we can see in these Tables, measurement noise certainly affects the overall performance of the ARU algorithm, where a higher noise tends to reduce the success rate and increase the number of iterations as well as that of the unique drug combinations to be tested. For many drug response functions considered in our simulations, the performance degradation is typically not too significant for the proposed algorithm, showing that the ARU algorithm is relatively robust to measurement noise. However, we can also observe that the extent of performance degradation will critically depend on the landscape of the underlying drug response. In most cases, the ARU algorithm continued to substantially outperform other stochastic search algorithms [9,11], demonstrating that it is better suited for practical drug optimization applications.

One interesting observation is that the performance of the Gur Game algorithm is typically not very sensitive to measurement noise. In fact, in some cases, its performance even improves as the noise level goes up. The main reason for this phenomenon is as follows. As discussed earlier, the Gur Game algorithm does not adapt to the observed drug response function, and for this reason, its overall performance crucially depends on whether or not its predetermined FSA matches the drug response function at hand. As a result, if the FSA does not match the original drug response function well, ironically enough, the measurement noise may perturb the search process in such a way that improves the overall performance. In this sense, the fact that the Gur Game algorithm is not very sensitive to measurement noise reflects its inaptitude for handling various types of drug response functions, rather than its robustness to random fluctuations and noise in the measured drug response.

## Conclusions

In this paper, we proposed a novel stochastic search algorithm, called the adaptive reference update (ARU) algorithm, which can be effectively used for optimizing the composition of combinatory drugs. The proposed algorithm intelligently utilizes the drug response values observed in the past to reliably predict how to beneficially update the drug concentrations to improve the drug response. As we demonstrated throughout this paper, the proposed algorithm addresses several shortcomings of previous drug optimization algorithms [9,11], thereby improving the overall search performance. Numerical experiments based on various types of multi-drug response functions show that the ARU algorithm results in a higher success rate (i.e., higher probability of identifying a potent drug combination) while requiring significantly fewer drug response evaluations. Furthermore, the proposed algorithm is robust to random measurement noise, where its search performance is not substantially affected in the presence of noise and degrades gracefully as the noise level increases. Throughout our experiments, we used *α *= 1 for the ARU algorithm as well as the previous search algorithm [11]. As discussed earlier, the parameter *α *controls the randomness of the search, by determining how much weight we should give to the drug response values that we observed in the past. In general, unless the observations are very noisy or the underlying drug response function is assumed to be extremely nonlinear, it would be best to set the parameter to the largest allowed value (i.e., *α *= 1), so that we fully utilize the past observations for making our best informed guess about the beneficial drug update strategy. For comparison, we also repeated our simulations using *α *= 0.5 and *α *= 0.75, whose results are summarized in Table S1 - Table S7 (see Additional file 1). We can see from these results that *α *= 1 indeed leads to the best performance for the drug response functions and the noise levels we have considered in this paper.

## Competing interests

The authors declare that they have no competing interests.

## Authors' contributions

Conceived and developed the algorithm: MK, BJY. Performed the experiments: MK. Analyzed the data and wrote the paper: MK, BJY.

## Supplementary Material

Additional file 1 **Performance of the ARU algorithm**. Further performance evaluation results of the proposed Adaptive Reference Update (ARU) algorithm. Click here for file
