# Supplementary material for: Adaptive reference update (ARU) algorithm. A stochastic search algorithm for efficient optimization of multi-drug cocktails
Source: BMC Genomics. 2012 Oct 26;13(Suppl 6):S12. doi: 10.1186/1471-2164-13-S6-S12 (PMC3488974; doi:10.1186/1471-2164-13-S6-S12)
Supplement: Additional file 1 — Performance of the ARU algorithm. Further performance evaluation results of the proposed Adaptive Reference Update (ARU) algorithm. [file 1471-2164-13-S6-S12-S1.pdf]

Supplementary Material

"Adaptive Reference Update (ARU) Algorithm: A Stochastic Search Algorithm for Efficient Optimization of Multi-Drug Cocktails"

Mansuck Kim[1] and Byung-Jun Yoon*[1]

[1] Department of Electrical and Computer Engineering, Texas A&M University, College Station, TX 77843-3128, USA

Email: Mansuck Kim - mk22893@neo.tamu.edu; Byung-Jun Yoon*- bjyoon@ece.tamu.edu;

*Corresponding author

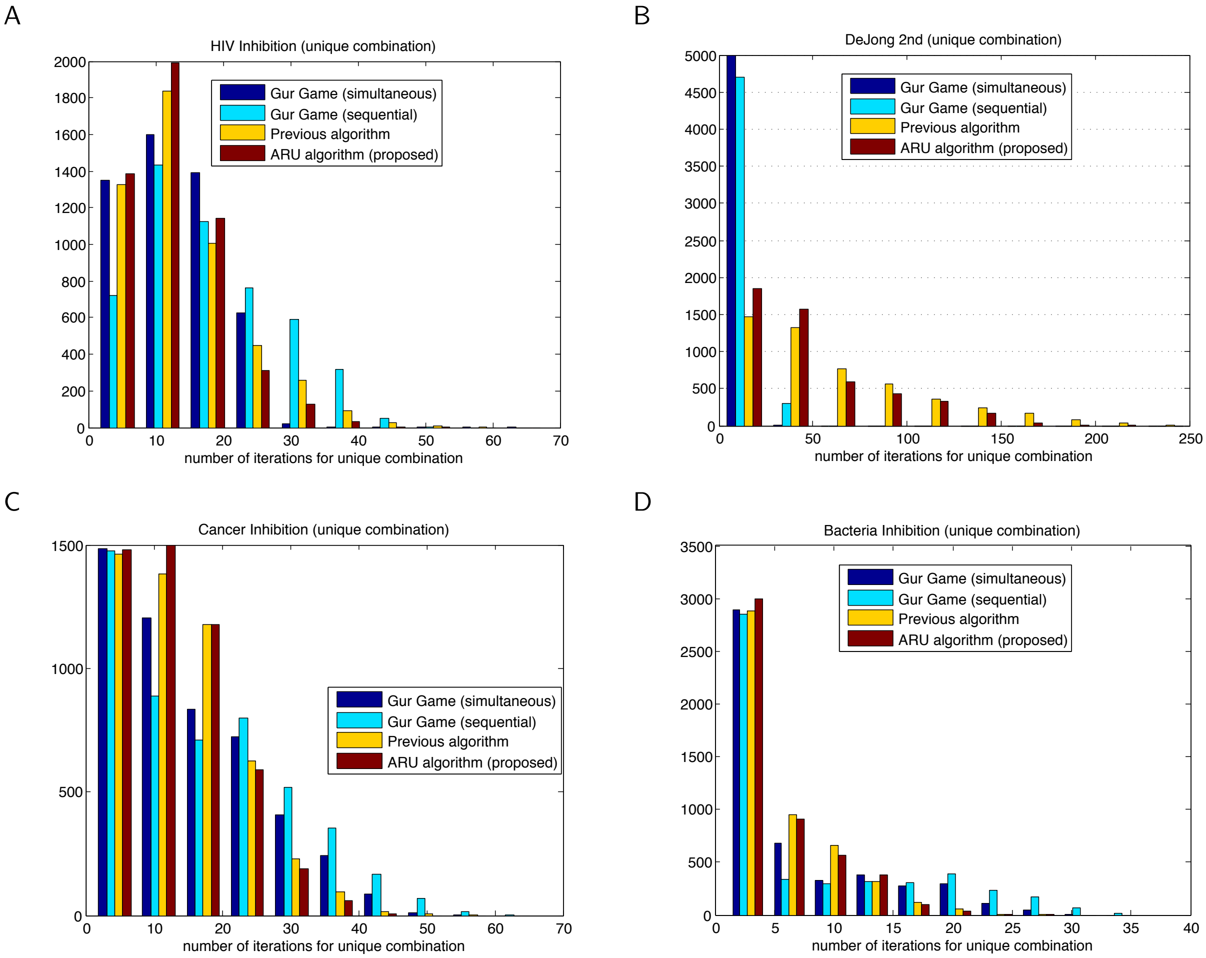

Figure S1: **Distribution of the number of unique drug combinations that need to be tested until an effective combination is identified.** (A) Inhibition of HIV. (B) Second De Jong function (Rosenbrock's saddle). (C) Inhibition of A549 lung carcinoma cell proliferation. (D) Inhibition of bacteria (*S. aureus*) proliferation.

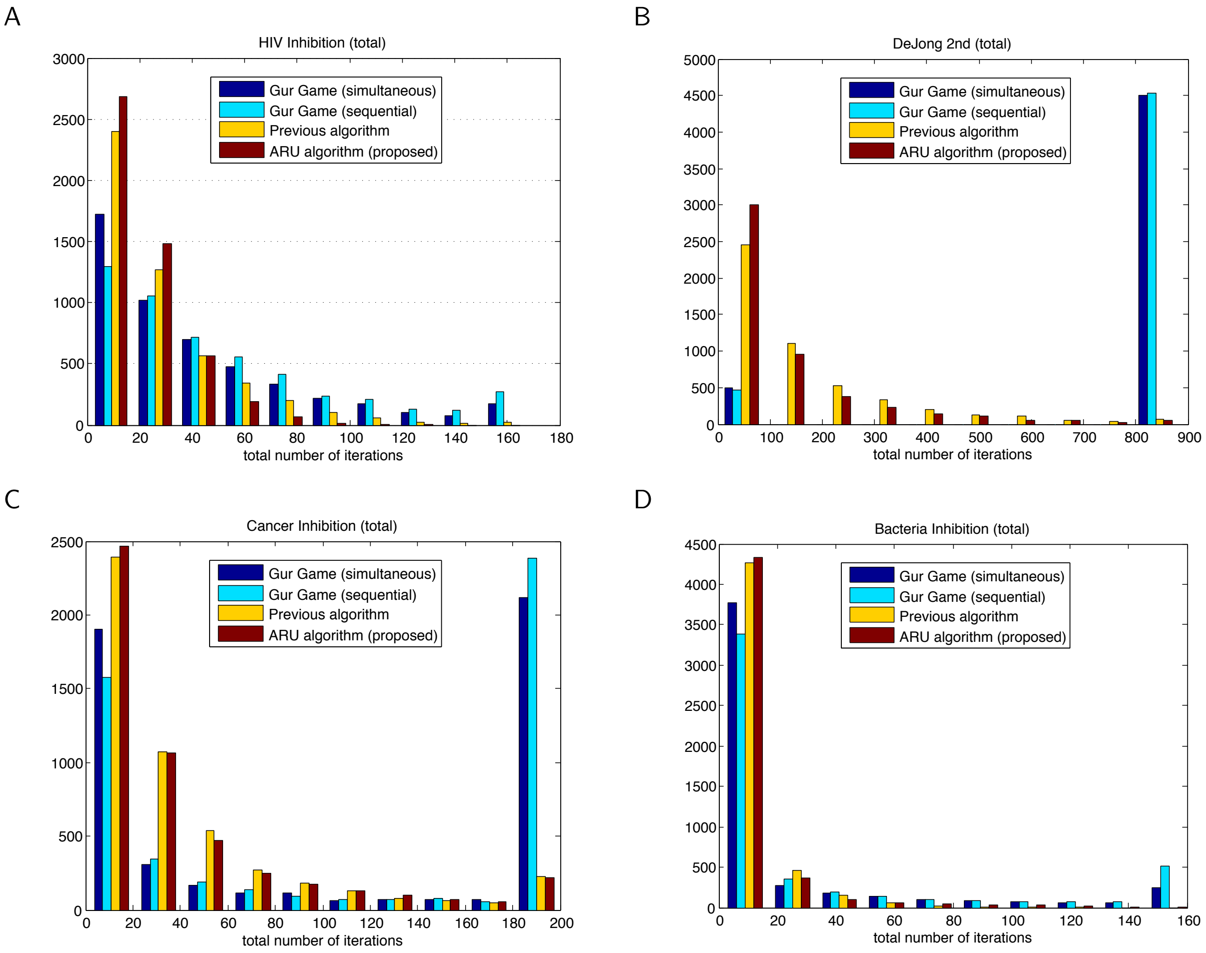

Figure S2: **Distribution of the number of search iterations that are needed until an effective combination is identified.** (A) Inhibition of HIV. (B) Second De Jong function (Rosenbrock's saddle). (C) Inhibition of A549 lung carcinoma cell proliferation. (D) Inhibition of bacteria (*S. aureus*) proliferation.

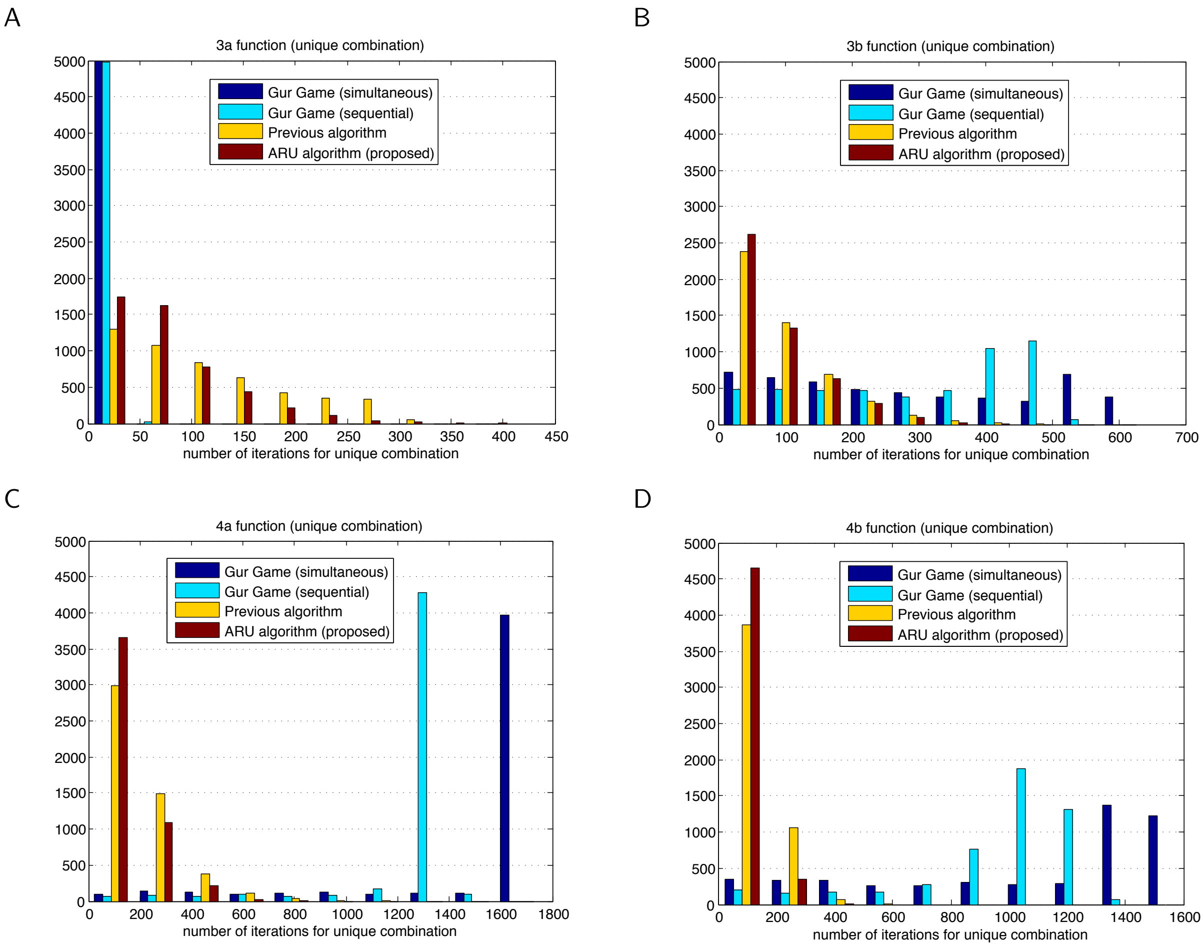

Figure S3: **Distribution of the number of unique drug combinations that need to be tested until an effective combination is identified.** (A) Three-dimensional drug response $f_{3a}(x_1, x_2, x_3)$. (B) Three-dimensional drug response $f_{3b}(x_1, x_2, x_3)$. (C) Four-dimensional drug response $f_{4a}(x_1, x_2, x_3, x_4)$. (D) Four-dimensional drug response $f_{4b}(x_1, x_2, x_3, x_4)$.

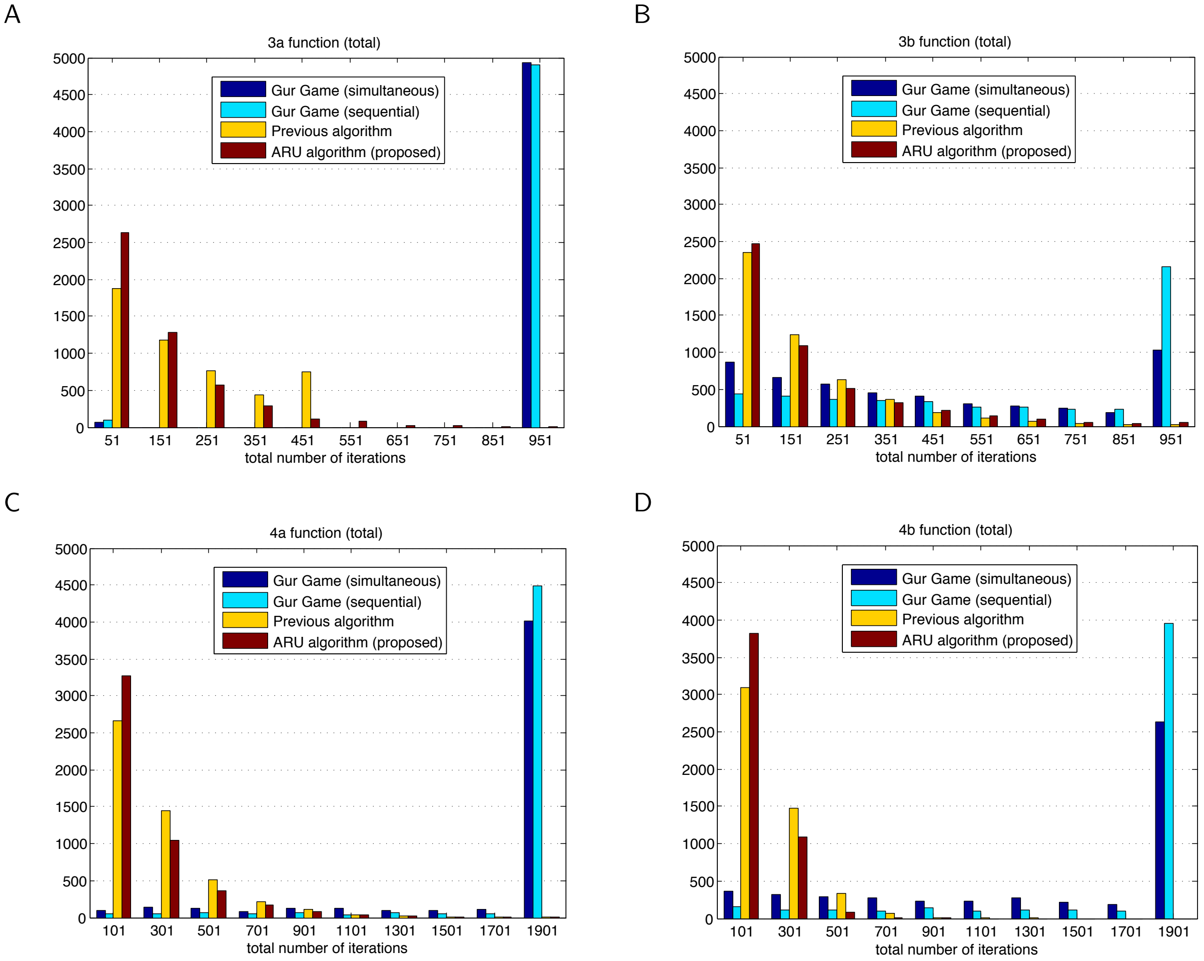

Figure S4: **Distribution of the number of search iterations that are needed until an effective combination is identified.** (A) Three-dimensional drug response $f_{3a}(x_1, x_2, x_3)$. (B) Three-dimensional drug response $f_{3b}(x_1, x_2, x_3)$. (C) Four-dimensional drug response $f_{4a}(x_1, x_2, x_3, x_4)$. (D) Four-dimensional drug response $f_{4b}(x_1, x_2, x_3, x_4)$.

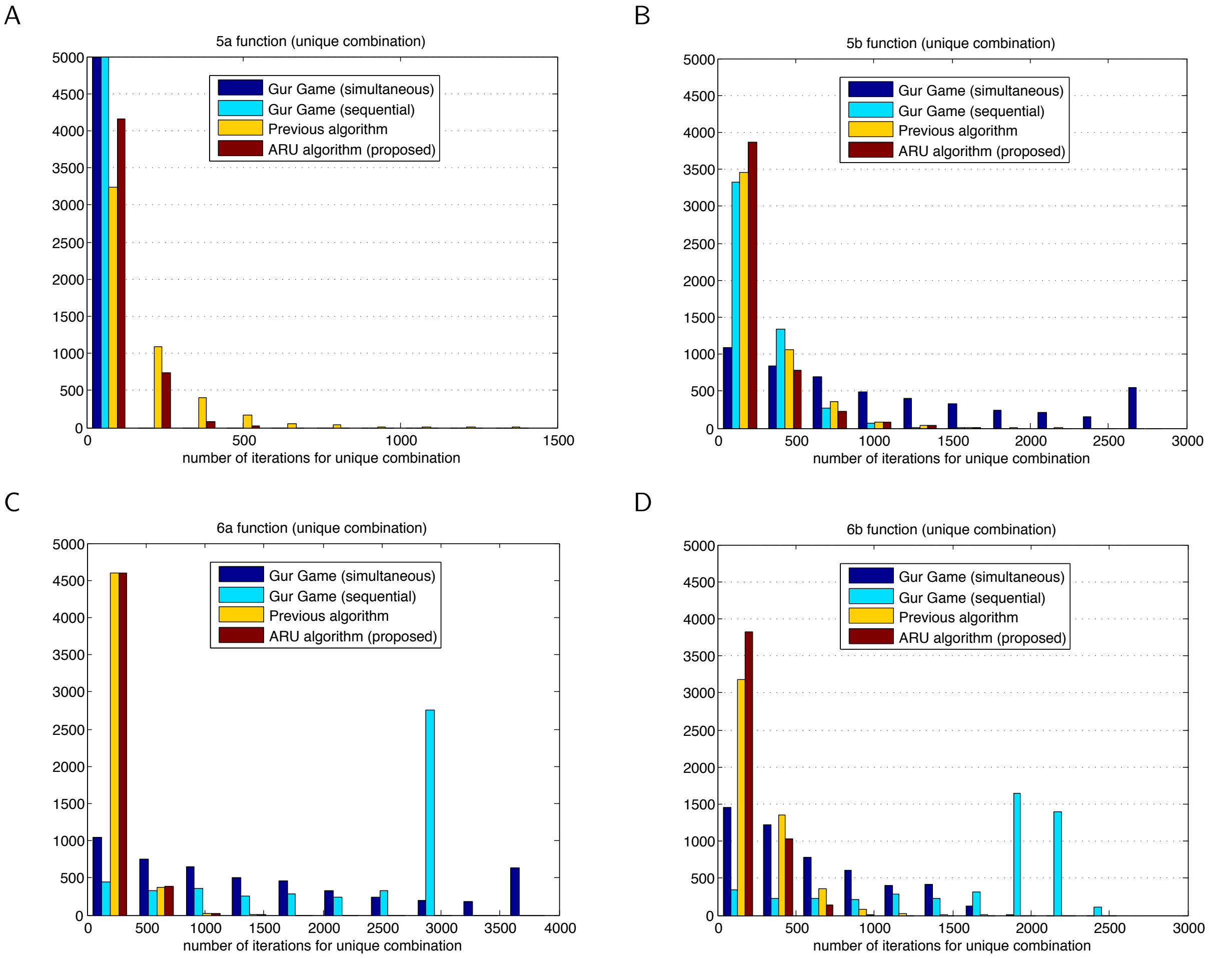

Figure S5: Distribution of the number of unique drug combinations that need to be tested until an effective combination is identified. (A) Five-dimensional drug response $f_{5a}(x_1, x_2, x_3, x_4, x_5)$. (B) Five-dimensional drug response $f_{5b}(x_1, x_2, x_3, x_4, x_5)$. (C) Six-dimensional drug response $f_{6a}(x_1, x_2, x_3, x_4, x_5, x_6)$. (D) Six-dimensional drug response $f_{6b}(x_1, x_2, x_3, x_4, x_5, x_6)$.

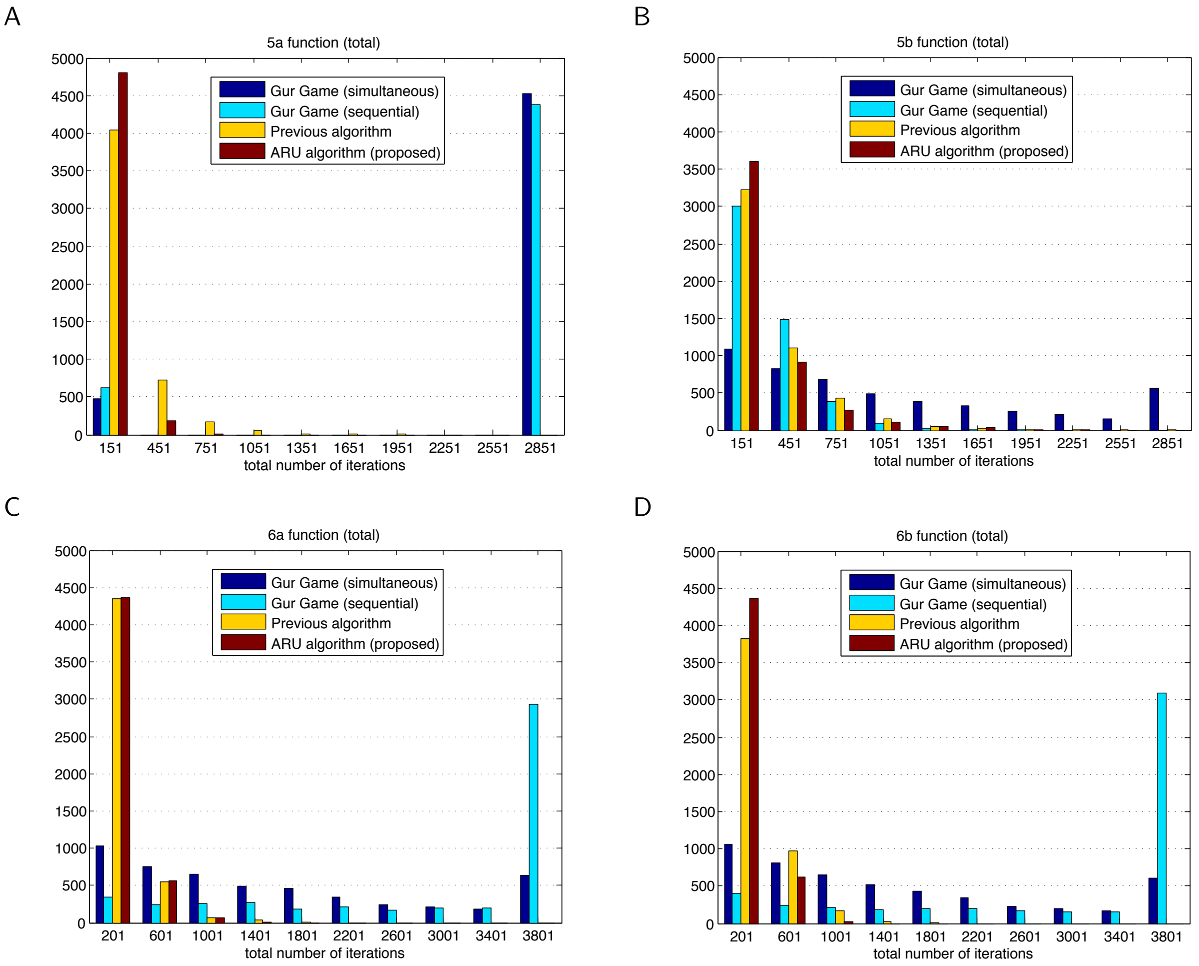

Figure S6: **Distribution of the number of search iterations that are needed until an effective combination is identified.** (A) Five-dimensional drug response $f_{5a}(x_1, x_2, x_3, x_4, x_5)$. (B) Five-dimensional drug response $f_{5b}(x_1, x_2, x_3, x_4, x_5)$. (C) Six-dimensional drug response $f_{6a}(x_1, x_2, x_3, x_4, x_5, x_6)$. (D) Six-dimensional drug response $f_{6b}(x_1, x_2, x_3, x_4, x_5, x_6)$.

Table S1: Performance for optimizing the combination of two drugs.

	Previous [11] ($\alpha = 0.5$)		Previous [11] ($\alpha = 0.75$)		ARU (proposed) ($\alpha = 0.5$)		ARU (proposed) ($\alpha = 0.75$)	
	success rate	unique comb.	success rate	unique comb.	success rate	unique comb.	success rate	unique comb.
$f_{2a}(\mathbf{x})$: HIV inhibition	97%	16.6	99%	15.0	100%	14.5	100%	13.0
$f_{2b}(\mathbf{x})$: De Jong (2nd)	96%	76.9	98%	64.7	99%	54.9	99%	49.1
$f_{2c}(\mathbf{x})$: Cancer inhibition	98%	16.7	98%	14.6	98%	15.5	98%	13.9
$f_{2d}(\mathbf{x})$: Bacteria inhibition	100%	5.4	100%	5.0	100%	5.1	100%	4.8

Table S2: Performance for optimizing multi-drug cocktails.

	Previous [11] ($\alpha = 0.5$)		Previous [11] ($\alpha = 0.75$)		ARU (proposed) ($\alpha = 0.5$)		ARU (proposed) ($\alpha = 0.75$)	
	success rate	unique comb.	success rate	unique comb.	success rate	unique comb.	success rate	unique comb.
$f_{3a}(\mathbf{x})$	100%	142.1	100%	122.4	100%	110.0	100%	85.6
$f_{3b}(\mathbf{x})$	96%	141.9	99%	109.5	99%	113.9	100%	92.0
$f_{4a}(\mathbf{x})$	90%	471.6	99%	299.8	99%	303.9	100%	188.9
$f_{4b}(\mathbf{x})$	92%	419.7	100%	220.1	100%	235.8	100%	136.1
$f_{5a}(\mathbf{x})$	100%	150.8	100%	147.1	100%	84.9	100%	82.7
$f_{5b}(\mathbf{x})$	99%	487.9	100%	345.0	100%	336.2	100%	256.6
$f_{6a}(\mathbf{x})$	100%	404.3	100%	249.3	100%	285.5	100%	226.1
$f_{6b}(\mathbf{x})$	100%	523.1	100%	344.7	100%	304.6	100%	223.6

Table S3: Performance for optimizing the combination of two drugs in the presence of noise.

	Noise level	Search type	Performance metric	Previous [11] ($\alpha = 0.5$)	Previous [11] ($\alpha = 0.75$)	ARU (proposed) ($\alpha = 0.5$)	ARU (proposed) ($\alpha = 0.75$)
$f_{2a}(\mathbf{x})$	**(2%)**	A	success rate	97%	99%	100%	100%
$f_{2a}(\mathbf{x})$	**(2%)**	A	unique comb.	16.4	14.8	14.3	12.8
$f_{2a}(\mathbf{x})$	**(2%)**	B	success rate	97%	99%	100%	100%
$f_{2a}(\mathbf{x})$	**(2%)**	B	iterations	38.6	31.1	27.1	22.6
$f_{2a}(\mathbf{x})$	**(5%)**	A	success rate	97%	99%	100%	100%
$f_{2a}(\mathbf{x})$	**(5%)**	A	unique comb.	16.4	14.8	14.3	12.9
$f_{2a}(\mathbf{x})$	**(5%)**	B	success rate	97%	99%	100%	100%
$f_{2a}(\mathbf{x})$	**(5%)**	B	iterations	38.6	31.7	27.5	22.9
$f_{2a}(\mathbf{x})$	**(8%)**	A	success rate	97%	99%	100%	100%
$f_{2a}(\mathbf{x})$	**(8%)**	A	unique comb.	16.8	14.8	14.3	13.0
$f_{2a}(\mathbf{x})$	**(8%)**	B	success rate	97%	99%	100%	100%
$f_{2a}(\mathbf{x})$	**(8%)**	B	iterations	39.5	32.9	28.2	23.8
$f_{2b}(\mathbf{x})$	**(2%)**	A	success rate	95%	97%	99%	99%
$f_{2b}(\mathbf{x})$	**(2%)**	A	unique comb.	76.0	63.1	55.5	49.1
$f_{2b}(\mathbf{x})$	**(2%)**	B	success rate	95%	98%	99%	99%
$f_{2b}(\mathbf{x})$	**(2%)**	B	iterations	218.0	175.7	148.1	126.6
$f_{2b}(\mathbf{x})$	**(5%)**	A	success rate	93%	96%	99%	99%
$f_{2b}(\mathbf{x})$	**(5%)**	A	unique comb.	81.4	70.9	61.7	56.5
$f_{2b}(\mathbf{x})$	**(5%)**	B	success rate	93%	97%	98%	99%
$f_{2b}(\mathbf{x})$	**(5%)**	B	iterations	230.7	205.2	177.3	153.7
$f_{2b}(\mathbf{x})$	**(8%)**	A	success rate	89%	94%	97%	98%
$f_{2b}(\mathbf{x})$	**(8%)**	A	unique comb.	82.9	73.5	69.3	61.9
$f_{2b}(\mathbf{x})$	**(8%)**	B	success rate	89%	96%	96%	98%
$f_{2b}(\mathbf{x})$	**(8%)**	B	iterations	259.5	221.8	196.1	178.0
$f_{2c}(\mathbf{x})$	**(2%)**	A	success rate	98%	98%	98%	98%
$f_{2c}(\mathbf{x})$	**(2%)**	A	unique comb.	16.8	14.7	15.6	14.0
$f_{2c}(\mathbf{x})$	**(2%)**	B	success rate	98%	98%	98%	98%
$f_{2c}(\mathbf{x})$	**(2%)**	B	iterations	42.2	37.1	39.1	37.6
$f_{2c}(\mathbf{x})$	**(5%)**	A	success rate	98%	98%	98%	98%
$f_{2c}(\mathbf{x})$	**(5%)**	A	unique comb.	16.3	14.5	14.7	13.6
$f_{2c}(\mathbf{x})$	**(5%)**	B	success rate	98%	98%	98%	98%
$f_{2c}(\mathbf{x})$	**(5%)**	B	iterations	42.7	37.7	39.6	37.8
$f_{2c}(\mathbf{x})$	**(8%)**	A	success rate	98%	98%	98%	98%
$f_{2c}(\mathbf{x})$	**(8%)**	A	unique comb.	15.3	13.7	14.1	13.0
$f_{2c}(\mathbf{x})$	**(8%)**	B	success rate	98%	98%	98%	98%
$f_{2c}(\mathbf{x})$	**(8%)**	B	iterations	43.5	38.5	40.5	38.3
$f_{2d}(\mathbf{x})$	**(2%)**	A	success rate	100%	100%	100%	100%
$f_{2d}(\mathbf{x})$	**(2%)**	A	unique comb.	4.9	4.5	4.6	4.3
$f_{2d}(\mathbf{x})$	**(2%)**	B	success rate	100%	100%	100%	100%
$f_{2d}(\mathbf{x})$	**(2%)**	B	iterations	9.5	8.7	8.7	8.3
$f_{2d}(\mathbf{x})$	**(5%)**	A	success rate	100%	100%	100%	100%
$f_{2d}(\mathbf{x})$	**(5%)**	A	unique comb.	4.8	4.4	4.6	4.3
$f_{2d}(\mathbf{x})$	**(5%)**	B	success rate	100%	100%	100%	100%
$f_{2d}(\mathbf{x})$	**(5%)**	B	iterations	9.6	8.8	8.5	8.1
$f_{2d}(\mathbf{x})$	**(8%)**	A	success rate	100%	100%	100%	100%
$f_{2d}(\mathbf{x})$	**(8%)**	A	unique comb.	4.8	4.4	4.5	4.4
$f_{2d}(\mathbf{x})$	**(8%)**	B	success rate	100%	100%	100%	100%
$f_{2d}(\mathbf{x})$	**(8%)**	B	iterations	9.6	8.8	8.4	7.9

Table S4: Performance for optimizing the combination of three drugs in the presence of noise.

	Noise level	Search type	Performance metric	Previous [11] ($\alpha = 0.5$)	Previous [11] ($\alpha = 0.75$)	ARU (proposed) ($\alpha = 0.5$)	ARU (proposed) ($\alpha = 0.75$)
$f_{3a}(\mathbf{x})$	**(2%)**	A	success rate	95%	98%	99%	100%
			unique comb.	147.9	128.6	112.6	89.0
		B	success rate	95%	98%	99%	100%
			iterations	290.9	243.6	198.6	163.9
	**(5%)**	A	success rate	95%	98%	99%	100%
			unique comb.	150.9	130.7	113.1	93.9
		B	success rate	95%	98%	99%	100%
			iterations	291.4	243.9	203.7	169.8
	**(8%)**	A	success rate	95%	98%	99%	100%
			unique comb.	153.0	132.9	116.6	97.3
		B	success rate	95%	98%	99%	100%
			iterations	291.8	244.7	210.1	178.4
$f_{3b}(\mathbf{x})$	**(2%)**	A	success rate	94%	98%	97%	98%
			unique comb.	155.2	129.6	130.4	112.8
		B	success rate	94%	98%	97%	99%
			iterations	304.6	240.1	253.1	221.4
	**(5%)**	A	success rate	92%	97%	96%	98%
			unique comb.	166.9	144.8	146.5	121.8
		B	success rate	91%	96%	95%	97%
			iterations	315.0	270.9	268.5	244.9
	**(8%)**	A	success rate	90%	96%	95%	97%
			unique comb.	172.4	147.9	152.9	130.2
		B	success rate	89%	94%	94%	97%
			iterations	324.5	281.3	293.7	263.6

Table S5: Performance for optimizing the combination of four drugs in the presence of noise.

	Noise level	Search type	Performance metric	Previous [11] ($\alpha = 0.5$)	Previous [11] ($\alpha = 0.75$)	ARU (proposed) ($\alpha = 0.5$)	ARU (proposed) ($\alpha = 0.75$)
$f_{4a}(\mathbf{x})$	**(2%)**	A	success rate	67%	86%	85%	95%
			unique comb.	578.9	509.1	507.3	398.1
		B	success rate	67%	85%	87%	97%
			iterations	826.9	689.8	711.0	578.4
	**(5%)**	A	success rate	59%	77%	76%	91%
			unique comb.	581.9	543.0	534.4	469.1
		B	success rate	61%	77%	76%	89%
			iterations	859.3	791.9	750.9	650.2
	**(8%)**	A	success rate	56%	70%	69%	84%
			unique comb.	606.0	566.6	592.8	507.7
		B	success rate	55%	71%	69%	84%
			iterations	883.7	844.4	840.9	736.9
$f_{4b}(\mathbf{x})$	**(2%)**	A	success rate	91%	100%	100%	100%
			unique comb.	440.4	241.2	268.4	143.9
		B	success rate	87%	99%	99%	100%
			iterations	698.9	417.3	471.3	261.3
	**(5%)**	A	success rate	78%	98%	98%	100%
			unique comb.	509.1	380.2	355.1	220.4
		B	success rate	71%	92%	94%	100%
			iterations	830.2	617.6	600.6	385.9
	**(8%)**	A	success rate	75%	88%	94%	99%
			unique comb.	576.7	436.4	411.2	291.2
		B	success rate	56%	79%	81%	97%
			iterations	879.2	793.5	753.8	560.6

Table S6: Performance for optimizing the combination of five drugs in the presence of noise.

	Noise level	Search type	Performance metric	Previous [11] ($\alpha = 0.5$)	Previous [11] ($\alpha = 0.75$)	ARU (proposed) ($\alpha = 0.5$)	ARU (proposed) ($\alpha = 0.75$)
$f_{5a}(\mathbf{x})$	**(2%)**	A	success rate	100%	100%	100%	100%
			unique comb.	144.7	142.2	129.2	125.7
		B	success rate	100%	100%	100%	100%
			iterations	184.4	182.3	164.1	157.2
	**(5%)**	A	success rate	100%	100%	100%	100%
			unique comb.	146.6	144.8	133.1	132.5
		B	success rate	100%	100%	100%	100%
			iterations	186.3	183.7	167.4	159.8
	**(8%)**	A	success rate	100%	100%	99%	100%
			unique comb.	150.2	146.5	137.8	134.4
		B	success rate	100%	100%	100%	100%
			iterations	188.6	185.4	170.2	162.2
$f_{5b}(\mathbf{x})$	**(2%)**	A	success rate	96%	98%	99%	100%
			unique comb.	696.3	544.1	547.8	438.8
		B	success rate	94%	99%	99%	100%
			iterations	831.9	692.7	649.9	539.6
	**(5%)**	A	success rate	93%	97%	96%	99%
			unique comb.	694.0	616.8	600.5	546.0
		B	success rate	93%	97%	97%	98%
			iterations	904.6	775.3	730.3	681.7
	**(8%)**	A	success rate	92%	95%	95%	97%
			unique comb.	718.2	661.5	634.2	605.3
		B	success rate	91%	95%	96%	98%
			iterations	955.8	834.3	783.8	748.9

Table S7: Performance for optimizing the combination of six drugs in the presence of noise.

	Noise level	Search type	Performance metric	Previous [11] ($\alpha = 0.5$)	Previous [11] ($\alpha = 0.75$)	ARU (proposed) ($\alpha = 0.5$)	ARU (proposed) ($\alpha = 0.75$)
$f_{6a}(\mathbf{x})$	**(2%)**	A	success rate	98%	99%	100%	100%
			unique comb.	685.3	573.8	636.4	549.6
		B	success rate	99%	100%	100%	100%
			iterations	876.4	647.9	710.6	614.2
	**(5%)**	A	success rate	96%	99%	98%	99%
			unique comb.	884.6	743.1	733.6	709.1
		B	success rate	97%	98%	98%	99%
			iterations	1026.2	838.6	892.1	809.0
	**(8%)**	A	success rate	94%	97%	96%	98%
			unique comb.	952.7	858.5	908.6	813.8
		B	success rate	95%	98%	97%	97%
			iterations	1126.8	988.1	1047.2	954.8
$f_{6b}(\mathbf{x})$	**(2%)**	A	success rate	99%	100%	100%	100%
			unique comb.	699.9	454.4	394.0	298.2
		B	success rate	99%	100%	100%	100%
			iterations	777.5	578.1	467.1	354.9
	**(5%)**	A	success rate	99%	100%	100%	100%
			unique comb.	724.5	544.3	451.8	348.5
		B	success rate	98%	100%	100%	100%
			iterations	927.2	633.2	544.9	418.2
	**(8%)**	A	success rate	99%	100%	100%	100%
			unique comb.	752.3	591.9	488.9	395.7
		B	success rate	98%	100%	100%	100%
			iterations	962.3	708.0	626.1	462.3
